# Trajectory and Correlation of Intrinsic Capacity and Frailty in a Beijing Elderly Community

**DOI:** 10.3389/fmed.2021.751586

**Published:** 2021-12-09

**Authors:** Shuo Liu, Lin Kang, XiaoHong Liu, SongQi Zhao, XuePing Wang, JiaoJiao Li, Shan Jiang

**Affiliations:** ^1^Department of Geriatrics, Peking Union Medical College, Peking Union Medical College Hospital, Chinese Academy of Medical Sciences, Beijing, China; ^2^Taikang Yanyuan Continuing Care Retirement Community, Beijing, China; ^3^Yanyuan Rehabilitation Hospital, Beijing, China; ^4^Department of Geriatrics, Affiliated Hospital of QingHai University, Xining, China; ^5^Department of Geriatrics, The Key Laboratory of Geriatrics, Beijing Institute of Geriatrics, Beijing Hospital, National Center of Gerontology, National Health Commission, Institute of Geriatric Medicine, Chinese Academy of Medical Sciences, Beijing, China

**Keywords:** frailty, intrinsic capacity, older adults, trajectory, transitions

## Abstract

**Objectives:** The World Health Organization proposed intrinsic capacity (IC) model to guide the implementation of person-centered care plan aimed at preserving or reserving functional ability, especially in frail older adults. We aimed to show the trajectory of IC and the overlap between IC impairment and frailty and investigate the correlation between IC domains and frailty status transitions.

**Method:** Longitudinal observational study covering 230 community-dwelling older adults (mean age 84.0 ± 4.5 years) at baseline, and transition information at 2-year follow-up (*n* = 196). IC was measured by five domains: locomotion, cognition, vitality, psychological, and sensory. Frailty was defined by FRAIL Scale. IC and frailty status transitions were assessed. Logistic regression, odds ratios (OR) and 95% confidence interval (CI) were used for the analysis.

**Results:** The prevalence of frailty was 23.0% and increased up to 41.8% over two years. Regarding frailty transitions, 38.3% of older adults progressed to more frailty status, and 8.6% regressed to lesser frailty status. The prevalence of IC impairment was 67.9% and increased to 81.6% over two years. Regarding IC transitions, 49.2% of adults with no IC impairment at baseline kept stable, and 50.8% developed new IC impairment. Among individuals with IC impairment at baseline, 57.9% worsened, and 13.5% improved. Importantly, IC impairment at baseline existed in 42.4% robust adults, 83.3% pre-frail adults, and 93.3% frail adults. 47.1% individuals who kept non-frail status within two years experienced IC worsened transition. Univariable analysis illustrated that new impaired locomotion, vitality, cognition, and sensory domains increased the risk of non-frail progressed to frail status. After adjusting for covariables, new impaired locomotion (OR = 3.625, 95% CI: 1.348–9.747) and vitality domains (OR = 3.034, 95% CI: 1.229–7.487) were associated with a higher possibility of non-frail progressed to frail status.

**Conclusion:** IC impairment and frailty overlap and co-exist in older adults. IC impairment, especially new impairment in locomotion and vitality are associated with the transitions from non-frail to frail status. It is important that geriatricians tightly monitor IC trajectory and find the new impaired domains to take early action to minimize the public health burden of frailty.

## Introduction

Population aging is accelerating rapidly worldwide, which brought a marked rise in the number of older adults with frailty (1). China is home to the largest population of older adults in the world (2), with the prevalence of frailty in older community-dwelling adults ranging from 5.9 to 17.4% (3). Previous relevant studies have revealed that higher age is related to higher frailty level (4). Frailty develops as the accumulated deficit in multiple physiological systems and is associated with an increased risk of poor outcomes like disability, falls, fracture, increased length of hospital stays, hospital readmission, hospital complications, morbidity, and mortality (5–7). According to several common frailty assessment tools, there is a universal description with three health statuses: robust, pre-frail, and frail. Based on these three statuses, much research exploring the trajectory of frailty demonstrates that it is a treatable and reversible clinical condition (8). Therefore, the detection of frailty should instead represent the entry point for more in-depth analysis with the aim of identifying the causes of an individual's increased vulnerability and implementing a person-centered care plan.

In order to overcome the weakness of frailty intervention and guide the implementation of health and social care plans in older community-dwelling adults, the World Health Organization (WHO) introduce intrinsic capacity (IC) to create a multidimensional construct related to individual's physical and mental ability (1, 9). IC can be evaluated by five domains: locomotion, cognition, vitality, psychological, and sensory capacities, reflecting the composite of all the physical and mental capacities (10, 11). Moreover, the WHO Healthy Aging model proposes that IC peaks in early adulthood and tends to decline from midlife onwards (12). Recent research demonstrated that IC can effectively predict adverse outcomes (e.g., falls and functional decline) in older community-dwelling adults (13, 14). Longitudinal studies over an extended period are needed to help us to investigate the extent to which the degree of IC changes as people age and guide person-centered care plan in older frail community-dwelling adults.

To our knowledge, the trajectory of IC with aging are still in its infancy and there are few studies to analyse the correlation between IC and frailty. In this study, we chose an elderly community with stable social support and a friendly living environment to show the natural trajectories of IC and frailty in order to avoid the interference of external factors. Moreover, we aimed to clarify the cross-sectional overlap between IC impairment and frailty and explore the impairment in IC domain and its correlation with frailty status transitions.

## Materials and Methods

### Study Design and Participants

The data used in this longitudinal observational cohort study was collected from a Beijing continuing care retirement community (CCRC) (15–17), which could provide friendly living environment, convenient medical services, and strong social support for the residents. The independent residents were assessed by community physicians before living in the CCRC active area. All the participants were recruited consecutively from June to August in 2018. The inclusion criteria were: (1) aged over 75 years old and (2) lived in the CCRC active area. The exclusion criteria for the participants were as follows: (1) acute conditions including acute heart failure, acute coronary syndrome, acute exacerbation of chronic obstructive pulmonary disease, and acute pneumonia, and (2) severe cognitive impairment diagnosed by a neurologist. Sociodemographic and clinical variables, such as age, sex, marital status, educational level, polypharmacy, and comorbidities were included. Polypharmacy was the numerical definition of five or more medications daily. Number and severity of comorbidities were evaluated with the Charlson Comorbidity index (CCI) (18). The Comprehensive Geriatric Assessment (CGA), including unplanned return visits, and hospitalisations due to acute and chronic diseases, and changes in social status (death of the partner) (19), was carried out by the experienced geriatricians at baseline (from June to August in 2018) and at 2-year follow-up (from August to September in 2020). All the geriatricians were from Peking Union Medical College Hospital (PUMCH). The participants provided their written informed consent to participate in this study. This study was approved by the Research Ethics Committee of Peking Union Medical College Hospital (PUMCH, JS2002).

### Measurements

#### Frailty and Frailty Status Transitions

Frailty status was defined based on FRAIL Scale (20) including five components: Fatigue (Do you feel tired at least 3 or 4 days per week?), Resistance (Can you climb one floor without assistance?), Ambulation (Can you walk one block or 100 m without assistance?), Illness (Do you suffer from more than five diseases?), and Loss of weight (Has your weight decreased by ≥4.5 kg or 5% of baseline in the previous 12 months?). Scores were assigned to each component (1 = Yes, 0 = No). Those who met 3–5 components were defined as frail, those with 1 or 2 components were deemed as pre-frail, and those without any were defined as robust. Participants who were robust or pre-frail were deemed as non-frail.

Frailty status transitions were classified into three categories: (1) Improved (adults who changed status from frail to non-frail), (2) Worsened (adults who changed status from non-frail to frail), and (3) Stable (adults with similar status at the follow-up period as the baseline status).

#### Intrinsic Capacity and IC Transitions

Intrinsic capacity (IC) was defined by five domains available from CGA Electronic Data Capture System. (1) Cognition was evaluated by Mini-Mental State Examination (MMSE) (21). Participants were cognitively impaired if they scored <25. (2) Psychological was assessed using the 15-item Geriatric Depression Scale (GDS-15) (22). The score ≥5 means psychological impairment. (3) Sensory (eye and hearing) was measured using two self-report questions. Do you have any difficulties in seeing far, reading, or eye diseases? Do you have any difficulties in hearing whisper? Both questions, if answered positively, means sensory dysfunction. (4) Vitality was evaluated with Mini Nutrition Assessment-Short form (MNA-SF) (23). The risk of malnutrition and malnutrition were deemed as vitality impairment. (5) Locomotion was measured by the Short Physical Performance Battery test (SPPB) (24). The sum score ≤ 9 means locomotion impairment. Each impaired domain is scored as 1 point, the total IC score is 5. An IC score ≥1 indicates IC impairment.

Intrinsic capacity (IC) transitions were defined based on changes of IC score at 2-year follow-up. Transitions were classified into three categories: (1) Improved (a decline of at least one point on IC score), (2) Worsened (an increase of at least one point on IC score), or (3) Stable (no change on IC score). Based on the 2-year difference in IC score (IC score at 2-year minus IC score at baseline), the 2-year change in number of impaired domains was identified.

### Statistical Analysis

Data are presented using descriptive statistics. The clinical characteristics of participants at baseline were described as mean (standard deviation) or median (interquartile range) for continuous variables and numbers with percentages (*n*, %) for categorical variables. The Chi-square test was conducted to compare categorical variables. The student's *t*-test or Mann-Whitney *U*-test was used to compare continuous variables between different groups. The frailty status transitions after 2-year follow-up were described using a Sankey-diagram (https://www.highcharts.com.cn/demo/highcharts/Sankey-diagram). Univariable and multivariable binary logistic regression analyses were used to evaluate the correlation between variables and frailty status transitions. For univariable analysis, each baseline characteristic was used as the independent variable and evaluated for its association with the dependent variable. We divided non-frail older adults into two groups according to the 2-year difference in IC score to identify statistically significant variables, as shown in the Supplementary Table 1. The covariables adjusted in multi-variable analysis were identified based on the statistically significant variables in univariable model. The odds ratios (OR) and 95% confidence intervals (CI) were reported. All statistical analysis was performed using SPSS (version 26.0; IBM SPSS Statistics for Windows, Armonk, NY: IBM Corporation). A *p*-value <0.05 was deemed as statistically significant.

## Results

### Baseline Characteristics of Study Population

Among 230 participants, 19 (8.3%) of them could not be contacted for follow-up due to moving back home, 15 participants were dead, and thus only 196 participants completed 2-year follow-up CGA. As shown in Table 1, there were no significant differences in age, sex, marital status, educational level, polypharmacy, and CCI between total participants (*n* = 230) and those who completed 2-year follow-up (*n* = 196). Among 196 participants, the mean age (SD) was 83.7 (4.4) years and 116 (59.2%) participants were female. The FRAIL Scale indicated a prevalence of pre-frail and frail of 33.7 and 23.0%, respectively. The median IC score was 1 (0–2). 133 (67.9%) participants were categorized as having IC impairment. The percentage of impairment in locomotion, cognition, vitality, psychological, and sensory domains were 58.2, 16.3, 14.3, 14.8, and 8.7%, respectively.

**Table 1:** Comparison of baseline characteristics between total sample and participants who completed follow-up in a Beijing elderly community.

**Baseline characteristics**	**Total participants (*n* = 230)**	**Participants who completed follow-up (*n* =196)**	***P*-value**
Age, mean (SD)	84.0 (4.5)	83.7 (4.4)	0.572
Female, *n* (%)	133 (57.8)	116 (59.2)	0.777
Marital status, *n* (%)			0.648
Married	106 (46.1)	86 (43.9)	
Divorced or windowed	124 (53.9)	110 (56.1)	
Educational level, *n* (%)			0.843
Below senior high school	3 (1.3)	3 (1.5)	
Senior high school or higher	227 (98.7)	193 (98.5)	
Polypharmacy, *n* (%)	130 (56.5)	110 (56.1)	0.934
CCI, median (IQR)	1 (0.2)	1 (0.2)	0.679

*CCI, Charlson Comorbidity Index; IQR, interquartile range; SD, standard deviation*.

### Frailty Status Transitions

**Figure 1:**
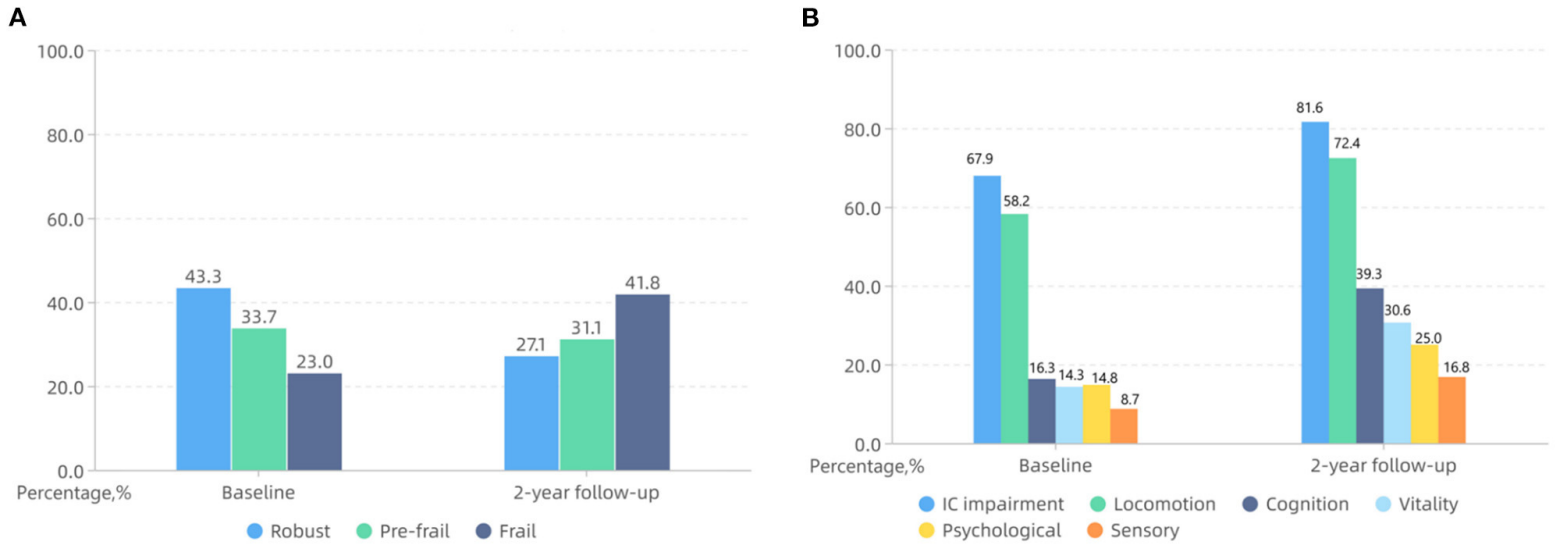
The percentage of frailty status **(A)**, IC impairment and impaired intrinsic capacity (IC) domains **(B)** at baseline and 2-year follow-up in a Beijing elderly community.

**Figure 2:**
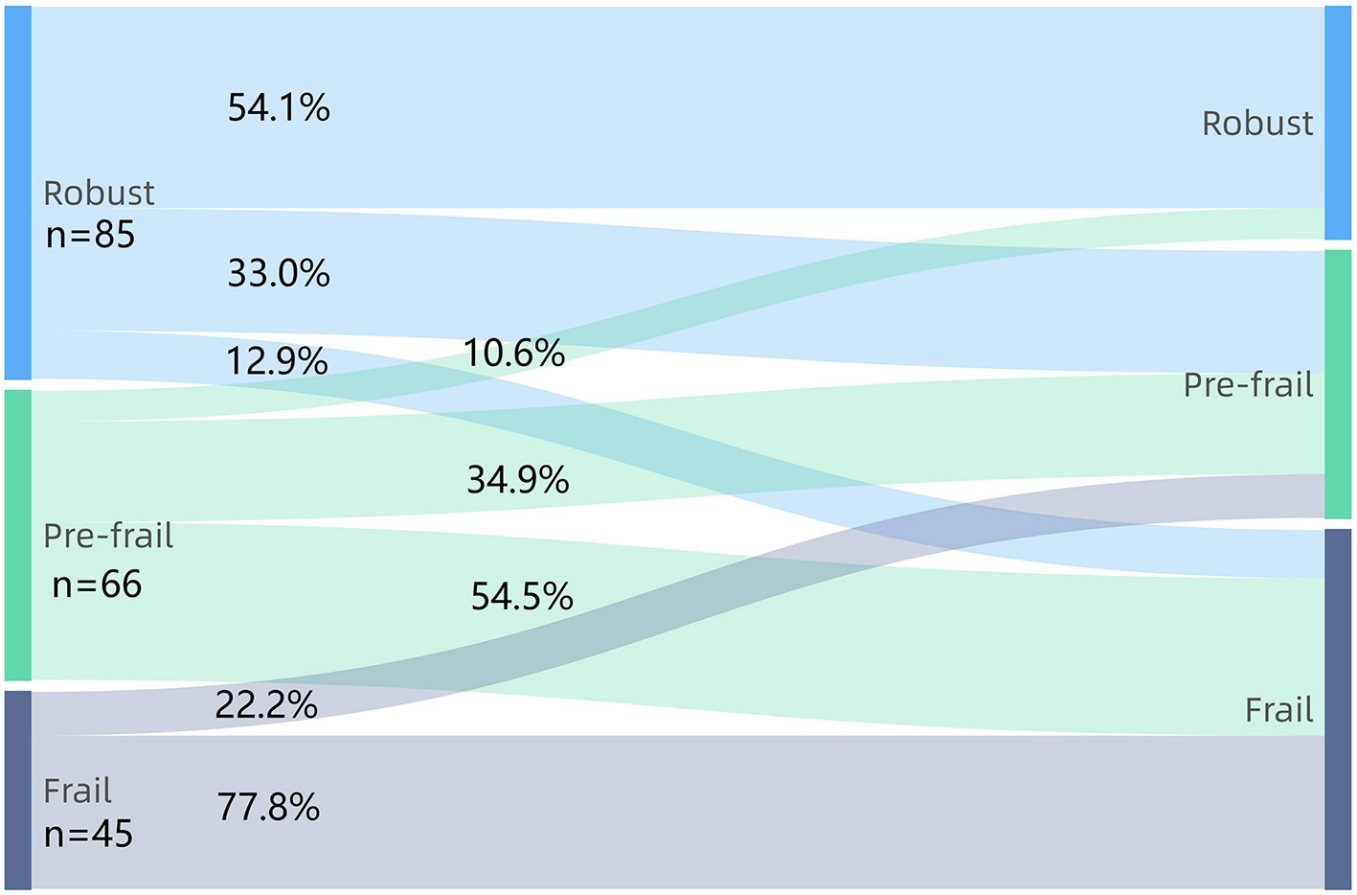
The frailty status transitions between baseline and 2-year follow-up.

The prevalence of frailty increased to 41.8% after two years, as shown in Figure 1. Figure 2 shows the frailty status transitions from the baseline to 2-year follow-up. 104 (53.1%) individuals maintained baseline status, and 92 (46.9%) made bidirectional transitions (75, 38.3% progressed, and 17, 8.6% regressed). Among 85 robust older adults at baseline, 33.0% became pre-frail, and 12.9% became frail. 54.5% of pre-frail adults at baseline progressed to frail status, and 10.6% of them regressed to robust older adults. Importantly, 22.2% of frail older adults regressed to pre-frail status and 77.8% still maintained frail. No frail older adults directly regressed to robust status. Among 47 non-frail adults who progressed to frail status, 14 (29.8%) experienced unplanned return visits, 19 (40.4%) experienced hospitalisations, and 4 (8.5%) had changes in social status (death of the partner). Lastly, only 15 (14.4%) experienced unplanned return visits, 25 (24.0%) suffered from hospitalisations, and none went through the death of partner among 104 adults who kept non-frail status during 2-year period.

### Intrinsic Capacity Transitions

The percentage of IC impairment was 81.6% with a median score of 2 (1–3) over two years. Regarding IC transitions, 69 (35.2%) individuals kept stable, and 127 (64.8%) made bidirectional transitions (109, 55.6% worsened, and 18, 9.2% improved). The 2-year difference of IC score ranged from −1 to 4 and the median (interquartile range, IQR) was 2 (0, 3). In the adults who experienced IC-worsened transitions, there were 38 (34.9%) individuals had unplanned return visits, 42 (38.5%) experienced hospitalisations, and 4 (3.7%) suffered from the death of partner. Among 63 participants with no IC impairment at baseline, 31 (49.2%) kept stable, and 32 (50.8%) worsened. For 133 participants with IC impairment at baseline, 18 (13.5%) of them improved, 38 (28.6%) kept stable, and 77 (57.9%) worsened. As shown in Figure 1, the overall rate of impairment in locomotion, cognition, vitality, psychological, and sensory domains increased to 72.4, 39.3, 30.6, 25.0, and 16.8%, respectively. Importantly, 49 (25.0%) participants developed new impairment in cognition domain. 42 (21.4%) individuals experienced new impairment in vitality domain, 30 (15.3%) individuals had new locomotion and psychological impairment, and only 19 (9.7%) adults showed new impairment in sensory domain.

### Correlation Between Intrinsic Capacity and Frailty Status Transitions

At baseline, IC impairment existed in 42.4% robust adults, 83.3% pre-frail adults, and 93.3% frail adults. After a 2-year period, IC impairment existed in 45.3% robust adults, 90.2% pre-frail adults, and 98.8% frail adults. According to frailty status transitions among non-frail adults, the comparison of characteristics between worsened and stable groups is shown in Table 2. Significant differences in age, polypharmacy, CCI, and new impairment in locomotion, vitality, cognition, and sensory domain were found between the two groups. No significant differences were reported about sex, marital status, and educational level between groups. Nearly half (47.1%) of adults who kept non-frail status within two years had experienced IC transitions worsened. Concerning non-frail older adults, the univariable analysis showed the 2-year change in the number of IC-impaired domains was correlated with frailty status transitions (OR = 2.125, 95% CI: 1.543–2.926, *P* < 0.001). The cut-off point of change in number of impaired domains for predicting frailty was 2. In multi-variables logistic regression analysis, the change in number of impaired domain (OR = 1.981, 95% CI: 1.424–2.756, *P* < 0.001) was also independently associated with frailty status transitions, after adjusting for age, polypharmacy, and CCI. Moreover, the univariable logistic regression analysis illustrated that age (OR = 1.142, 95% CI: 1.046–1.246), polypharmacy (OR = 2.797, 95% CI: 1.354–5.779), CCI (OR = 1.749, 95% CI: 1.237–2.471), and new impaired locomotion (OR = 2.676, 95% CI: 1.128–6.349), vitality (OR = 2.839, 95% CI: 1.269–6.348), cognition (OR = 2.262, 95% CI: 1.079–4.740), and sensory domain (OR = 4.062, 95% CI: 1.251–13.181) increased the risk of non-frail adults progressed to frail status (*P* < 0.05). After adjusting for age, polypharmacy, and CCI, the results showed that new impaired locomotion (OR = 3.625, 95% CI: 1.348–9.747) and vitality domain (OR = 3.034, 95% CI: 1.229–7.487) were associated with a higher probability of non-frail status progressed to frail status as given in Table 3.

**Table 2:** Comparison of characteristics among non-frail and frail participants according to frailty status transitions in a Beijing elderly community.

	**Non-frail (*****n*** **= 151)**			**Frail (*****n*** **= 45)**		
**Characteristics**	**Worsened (*n* = 47)**	**Stable (*n* = 104)**	***P*-value**	**Stable (*n* = 35)**	**Improved (*n* = 10)**	***P*-value**
Age, mean (SD)	84.7 (4.0)	82.4 (4.3)	0.002	85.8 (4.3)	85.4 (2.4)	0.838
Female, *n* (%)	26 (55.3)	58 (55.8)	0.959	25 (69.4)	8 (88.9)	0.238
Marital status, *n* (%)			0.382			
Married	19 (40.4)	50 (48.1)		14 (40.0)	3 (30.0)	0.565
Divorced or windowed	28 (59.6)	54 (51.9)		21 (60.0)	7 (70.0)	
Educational level, *n* (%)			NA			0.632
Below senior high school	0	0		2 (5.7)	1 (10.0)	
Senior high school or higher	47	104		33 (94.3)	9 (90.0)	
Polypharmacy, *n* (%)	32 (68.1)	45 (43.3)	0.005	26 (74.3)	7 (70.0)	0.787
CCI, median (IQR)	1 (0–2)	0 (0–1)	0.009	1 (1–2)	1 (1–3)	0.308
New impaired domains, *n* (%)						
Locomotion	13 (27.7)	13 (12.5)	0.022	4 (11.4)	0	0.263
Vitality	16 (34.0)	16 (15.4)	0.009	9 (25.7)	1 (10.0)	0.292
Cognition	19 (40.4)	24 (23.1)	0.029	5 (14.3)	1 (10.0)	0.725
Psychological	9 (19.1)	12 (11.5)	0.211	9 (25.7)	0	0.073
Sensory	8 (17.0)	5 (4.8)	0.013	5 (14.3)	1 (10.0)	0.725
IC transition, *n* (%)			0.000			0.269
Improved	0	11 (10.6)		5 (14.3)	2 (20.0)	
Stable	6 (12.8)	44 (42.3)		13 (37.1)	6 (60.0)	
Worsened	41 (87.2)	49 (47.1)		17 (48.6)	2 (20.0)	

*BMI, body mass index; CCI, Charlson Comorbidity Index; IC, intrinsic capacity; IQR, interquartile range; NA, not available; SD, standard deviation*.

**Table 3:** Logistic regression analysis showing the correlation between new impaired domains and frailty status transitions in non-frail older adults.

**New impaired domains**	**Uni-variable**		**Multi-variable^a^**	
	**OR (95% CI)**	***P*-value**	**OR (95% CI)**	***P*-value**
Locomotion	2.676 (1.128–6.349)	0.025	3.625 (1.348–9.747)	0.011
Cognition	2.262 (1.079–4.740)	0.031	1.960 (0.878–4.375)	0.101
Vitality	2.839 (1.269–6.348)	0.011	3.034 (1.229–7.487)	0.016
Psychological	1.816 (0.707–4.664)	0.215	1.650 (0.596–4.565)	0.335
Sensory	4.062 (1.251–13.181)	0.020	3.400 (0.922–12.537)	0.066

*OR, odds ratio; CI, confidence interval*;

a *including age, polypharmacy and Charlson Comorbidity Index*.

For frail older adults at baseline, no differences of characteristics were observed between stable and improved groups as shown in Table 2, neither in the univariable and multivariable analysis.

## Discussion

The present study investigated the natural trajectories of IC and frailty status, the main changes of IC domain, and its correlation with frailty status transitions in community-dwelling older adults. This is of clinical and public health interest since little is known regarding IC trajectories within 2-year period and the influence on frailty status transitions.

### The Trajectory of Frailty Status and Intrinsic Capacity

We found that the prevalence of frailty according to FRAIL Scale was 23.0%. The result is consistent with the finding from a meta-analysis, which found that the prevalence of frailty among Chinese community-dwelling older adults aged over 80 years was 21.6% (25). Moreover, the prevalence of frailty at 2-year period was considerably higher than the baseline. This is in line with previous studies based on different frailty assessment tools wherein frailty is correlated with age (25, 26). This longitudinal observational study also proved that frailty is a dynamic process, which was comparable with previous research (27, 28). Half of older adults remained stable, with pre-frail adults being more likely to become frail than robust adults. Importantly, older adults can regress into lesser frailty status, although the probability is low. Thus, the early development and implementation of comprehensive person-centered care plan aimed at preserving or reserving frailty brook no delay.

Intrinsic capacity (IC) impairment was present in 67.9% of the community-dwelling older adults. The locomotion and cognition were the two most vulnerable domains in IC model. This result is in agreement with early findings which showed the percentage of IC impairment ranged from 69.1 to 75.3% (29, 30). It is almost certain that IC impairment is quite common among community-dwelling older adults. Another important finding was that the prevalence of IC impairment increased to 81.6% over two years, and the percentages of impaired domains also increased. This is also in accordance with the IC model that individuals' capacities will decline with aging (31). In addition, in IC trajectories, cognition and vitality were the two domains most prone to new impairment. At an individual level, while trajectories are continuous, they are rarely smooth. There is great variability in these trajectories and some components of capacity may remain stable, decrease, or even increase over the life course. Therefore, tracing IC trajectories can inform us to take action to reverse the trend and inform the effectiveness of the interventions implemented or the variation in one's needs.

Moreover, the occurrence of unplanned return visits and hospitalisations due to acute and chronic disease and changes in social status (death of the partner) were common in the older adults who experienced frailty status from non-frail or IC worsened transitions during 2-year period. There are two likely causes for the results. On one hand, frailty and IC decline can increase the risk of adverse health outcomes (e.g., hospitalisations, fall, and functional decline) in older adults. On the other hand, older adults may suffer from hospital-acquired complications, which cause the worsened transitions of frailty status or IC. Therefore, they may form a vicious circle, with each condition affecting the other.

### IC Worsened Transition Occurs in Non-Frail Adults and New Impaired Locomotion and Vitality Predict Frail Status

The empirical results in this study provide a new understanding of IC impairment overlap with frailty status. Among robust older adults, nearly half of them existed IC impairment. The most interesting finding was that non-frail adults had experienced IC-worsened transition, even though they kept non-frail status over two years. Our study demonstrated that many individuals will experience a significant IC decline that is associated with frailty before death. In accordance with previous work, the WHO model of Healthy Aging proposes that this period of significant loss of IC is often preceded by earlier, more robust, health states (12, 32). Another cohort study from Gutierrez-Robledo and collaborators also demonstrate that IC is significantly associated in a cross-sectional way with frailty (33). We found that two or more impaired domain in 2-year follow-up period was associated with worsened frailty status among non-frail adults. Therefore, the monitoring of IC trajectories can support the detection of the individuals' fragilization. In the present study, there was no variability of educational level between stable and worsened groups among non-frail older adults. The reasons might be related to the selectivity of the study sample. All the participants lived in the CCRC active area with excellent living environment and social support, and most of them had good educational experience. In addition, significant differences in new impaired locomotion, vitality, cognition, and sensory domains were founded between stable and worsened groups. After adjusting for co-variables, new impairment in locomotion and vitality domains had the predictive value for frailty status among non-frail older adults over two years. Thus, from the perspective of IC, exercise and nutritional supplementation prevail as mainstays of person-centered care interventions. This study produced results that corroborate the findings of a great deal of the previous work in the frailty intervention field (34).

Surprisingly, no differences in IC domains were found between stable and improved groups among baseline frail older adults. The observed correlation might be explained in this way. Frailty is the long-term outcome of IC impairment in older age, and frail older adults are characterized by significant losses of IC. Previous study also described that frailty and IC were two distinct but related constructs, stemming from the same need of overcoming traditional medical paradigms (35, 36). Taking a more holistic life course approach, frailty is a state of reduced resilience to stressful events that occurs in response to physiological and/or psychosocial detriments (37, 38). Therefore, frail older adults have less reversibility than non-frail adults. Frailty is potentially preventable up to a probable point of no return when it becomes a pre-death phase. It is challenging to improve IC to maintain less frailty status. Collectively, the interventions must be prioritized in ways that optimize trajectories of physical and mental capacities to avoid the onset and development of frailty, especially in non-frail adults.

The main strength of this research was the study was conducted in the CCRC, which is characterized by similar nutritional supplements and stable surrounding environment. Our results reflected the natural trajectories of IC and frailty within a two-year period in an elderly community. In addition, the changes of each IC domain and its relationship with frailty status transitions were shown in our study. However, there were some limitations in our study. First, our sample size was not big enough and further multi-center studies are needed to prove the results. Second, our study was not designed to measure IC in advance. All items were spared from CGA, but consistent with IC domains proposed by WHO. Third, FRAIL Scale used in our study was a self-report scale, which may affect the judgment of frailty status. Objective measurements may increase the accuracy of the results and reduced the overestimation or underestimation of frailty status. However, previous research has demonstrated that FRAIL Scale is a time and cost-effective screening tool with good validity and diagnostic test accuracy.

## Conclusion

To the best of our knowledge, there were few longitudinal observational studies based on community-dwelling older adults to reveal the natural trajectories of IC and frailty simultaneously. Our results demonstrate the dynamics and diversity of IC with age and show IC impairment and frailty overlap and co-exist in older adults. It is important that geriatricians tightly monitor the natural IC trajectory and the new impairment in domains, to take early preventive and rehabilitative actions to minimize the public health burden of frailty.

## Data Availability Statement

The raw data supporting the conclusions of this article will be made available by the authors, without undue reservation.

## Ethics Statement

The studies involving human participants were reviewed and approved by the Research Ethics Committee of Peking Union Medical College Hospital (PUMCH, JS2002). The patients/participants provided their written informed consent to participate in this study.

## Author Contributions

SL, LK, and XL contributed to the design of the work, contributed to the analysis, and interpretation of data. SL, SZ, XW, JL, and SJ contributed to the data collection. SL drafted the manuscript. All authors contributed to writing the paper and revising it critically and gave final approval of this version.

## Funding

This work was supported by National Key R&D Program of China (2018YFC2002100 and 2018YFC2002104) and CAMS Innovation Fund for Medical Sciences (CIFMS) (2018-I2M-1-002).

## Conflict of Interest

The authors declare that the research was conducted in the absence of any commercial or financial relationships that could be construed as a potential conflict of interest.

## Publisher's Note

All claims expressed in this article are solely those of the authors and do not necessarily represent those of their affiliated organizations, or those of the publisher, the editors and the reviewers. Any product that may be evaluated in this article, or claim that may be made by its manufacturer, is not guaranteed or endorsed by the publisher.
