# Supplementary material for: Trajectory and Correlation of Intrinsic Capacity and Frailty in a Beijing Elderly Community
Source: Front Med (Lausanne). 2021 Dec 9;8:751586. doi: 10.3389/fmed.2021.751586 (PMC8695757; doi:10.3389/fmed.2021.751586)
Supplement: Supplementary file 1 [file Table_1.DOCX]

Supplementary Material

Supplementary Table 1. Comparsion of baseline characteristics among 147 non-frail older adults according to 2-year difference in IC score (the number of impaired domains)

| Variables | 2-year difference in IC score ≤ 2 (n=103) | 2-year difference in IC score>2 (n=48) | P value |
| --- | --- | --- | --- |
| Age,mean(SD) | 82.5(4.2) | 84.5(4.3) | 0.016 |
| Female,n(%) | 58(56.3) | 26(54.2) | 0.806 |
| Marital status,n(%) |  |  |  |
| Married | 49(476) | 20(41.7) | 0.499 |
| Divorced or widowed | 54(52.4) | 28(58.3) |  |
| Educational level,n(%) |  |  | NA |
| Below senior high school | 0 | 0 |  |
| Senior high school or higher | 103 | 48 |  |
| Polypharmacy,n(%) | 47(45.6) | 30(62.5) | 0.050 |
| CCI,Median(IQR) | 0(0,1) | 1(0,2) | 0.021 |
